# Association of intrapartum epidural analgesia and oxytocin exposure with offspring autism and neurodevelopment from the Japan Environment and Children’s Study

**DOI:** 10.1038/s41598-026-46497-8

**Published:** 2026-05-18

**Authors:** Asuka Tachi, Yuki Takahashi, Yuki Ito, Sayaka Kato, Mayumi Sugiura-Ogasawara, Shinji Saitoh, Michihiro Kamijima, the Japan Environment, Children’s Study Group, Shin Yamazaki, Maki Fukami, Reiko Kishi, Chiharu Ota, Koichi Hashimoto, Kenichi Sakurai, Shuichi Ito, Ryoji Shinohara, Hidekuni Inadera, Takeo Nakayama, Ryo Kawasaki, Yasuhiro Takeshima, Hideki Nagashima, Narufumi Suganuma, Mayumi Tsuji, Kimitoshi Nakamura

**Affiliations:** 1 Department of Occupational and Environmental Health, Graduate School of Medical Sciences, Nagoya City University, 1 Kawasumi, Mizuho-cho, Mizuho-ku, Nagoya, 467-8601 Japan; 2 School of Nursing, Kyoto Prefectural University of Medicine, 465 Kajiichō, Kawaramachi-dōri Hirokoji-Agaru, Kamigyō-ku, Kyoto, Japan; 3 Department of Obstetrics and Gynecology, Graduate School of Medical Sciences, Nagoya City University, 1 Kawasumi, Mizuho-cho, Mizuho-ku, Nagoya, 467-8601 Japan; 4 Department of Pediatrics and Neonatology, Graduate School of Medical Sciences, Nagoya City University, 1 Kawasumi, Mizuho-cho, Mizuho-ku, Nagoya, 467-8601 Japan; 5 National Institute for Environmental Studies, Tsukuba, Ibaraki Japan; 6 National Center for Child Health and Development, Tokyo, Japan; 7 Hokkaido University, Sapporo, Hokkaido Japan; 8 Tohoku University, Sendai, Miyagi Japan; 9 Fukushima Medical University, Fukushima, Japan; 10 Chiba University, Chiba, Japan; 11 Yokohama City University, Yokohama, Kanagawa Japan; 12 University of Yamanashi, Chuo, Yamanashi Japan; 13 University of Toyama, Toyama, Japan; 14 Kyoto University, Kyoto, Japan; 15 Osaka University, Suita, Osaka Japan; 16 Hyogo Medical University, Nishinomiya, Hyogo Japan; 17 Tottori University, Yonago, Tottori, Japan; 18 Kochi University, Nankoku, Kochi Japan; 19 University of Occupational and Environmental Health, Kitakyushu, Fukuoka Japan; 20 Kumamoto University, Kumamoto, Japan

**Keywords:** Autism spectrum disorder, Labor epidural analgesia, Oxytocin exposure, Neurodevelopment, Longitudinal cohort study, Japan Environment and Children’s Study (JECS), Health care, Medical research, Neurology, Neuroscience, Risk factors

## Abstract

Given the increasing prevalence of neurodevelopmental disorders, this study examined the association between autism and neurodevelopmental delays and exposure to intrapartum labor epidural analgesia (LEA) and synthetic oxytocin (OT) in offspring. The data were obtained from various questionnaires, including the Japanese Ages and Stages Questionnaires, Third Edition. Using data from a large-scale longitudinal birth cohort study in Japan, 72,801 participants were enrolled and follow-up to age 4 years. Exposure during labor was categorized into four groups: no-exposure, OT, LEA, and LEA-OT. Adjusted odds ratios for autism were 2.35 (95% CI 1.44–3.83) in the LEA-OT group and 2.41 (95% CI 1.31–4.45) in the LEA group. The E-values for the point estimates were 4.16 in the LEA-OT group and 4.29 in the LEA group, and those for the lower limits of the 95% confidence intervals were 2.39 and 2.05, respectively, indicating only moderate robustness to unmeasured confounding. Associations with autism were consistent across analyses in the LEA-OT group, but in sex-stratified analyses, only males showed an association. No consistent associations with neurodevelopmental delays were observed in any exposure groups. Since residual confounding cannot be excluded, with limited external validity, these findings warrant replication in independent cohorts to clarify causality.

**Supplementary Information:**

The online version contains supplementary material available at 10.1038/s41598-026-46497-8.

## Introduction

Increasing incidence of neurodevelopmental disorders, such as autism spectrum disorder (ASD)^1^ and attention-deficit/hyperactivity disorder^1,2^, is a global health concern that cannot be explained solely by advanced diagnostic techniques and increased awareness^3–5^. Epidemiological studies have reported perinatal risk factors for neurodevelopmental disorders, including gestational diabetes^6^, advanced parental age^7^, preterm birth^8^-as well as labor interventions, such as labor epidural analgesia (LEA) or synthetic oxytocin (OT) administration^9–14^.

LEA, which is recommended for labor pain relief, is used in 19–83% of nulliparous women and 10–64% of multiparous women in high‑income countries^15,16^. In Japan, the proportion of labor analgesia increased from 2.6% in 2009 to 11.6% in 2023^17,18^. No adverse neonatal outcomes have been demonstrated; however, data on long-term outcomes remain limited^19,20^. While associations between LEA and ASD or developmental delays have been noted, findings remain inconsistent^9–11,14,21–23^.

Endogenous OT promotes intrapartum uterine contractions, milk ejection, and mother–child attachment^24^. Synthetic OT is widely used to induce and augment labor, with rates exceeding 30% of women in many low- and lower-middle-income countries and in about one‑fifth of women in a large nationwide Japanese birth cohort^25,26^. Studies on neonatal outcomes with synthetic OT exposure during labor have yielded some results regarding their associations with low Apgar scores, increased neonatal intensive care unit admissions, instrumental delivery rates, and cesarean sections and altered breast-seeking behavior^27–29^. Long‑term effects of oxytocin administration for labor induction or augmentation on neurodevelopment and ASD have been the focus of recent consensus^12,13,21,26,30,31^, but further studies are still needed.

LEA is frequently used with oxytocin, as it reduces uterine contractility and prolongs the duration of labor^32^. As such, the need for OT is more than double with LEA^33^. A recent review reported an association between combined use of LEA and OT during labor and offspring outcomes^34^; however, the results were limited to including offspring outcomes such as neonatal behavior, skin temperature patterns, neonatal jaundice, and ASD^21,35–37^. Furthermore, most of these studies were limited by small sample sizes and observational designs, yielding inconsistent results^34^. Therefore, larger cohort studies focusing on several aspects of offspring neurodevelopment are warranted to evaluate the long-term developmental effects of intrapartum LEA and OT administration.

Therefore, this study investigated the association between the combined use of LEA and OT during labor and offspring ASD and neurodevelopment outcomes by categorizing intrapartum exposure into four groups: No-exposure (exposure to neither OT nor LEA), OT (exposure to OT), LEA (exposure to LEA), and LEA-OT (exposure to both LEA and OT). While the combination of LEA and OT is widely used based on clinical needs and guidelines, these findings may provide useful information for healthcare providers and mothers considering its use.

## Results

### Participant characteristics

**Fig. 1:**
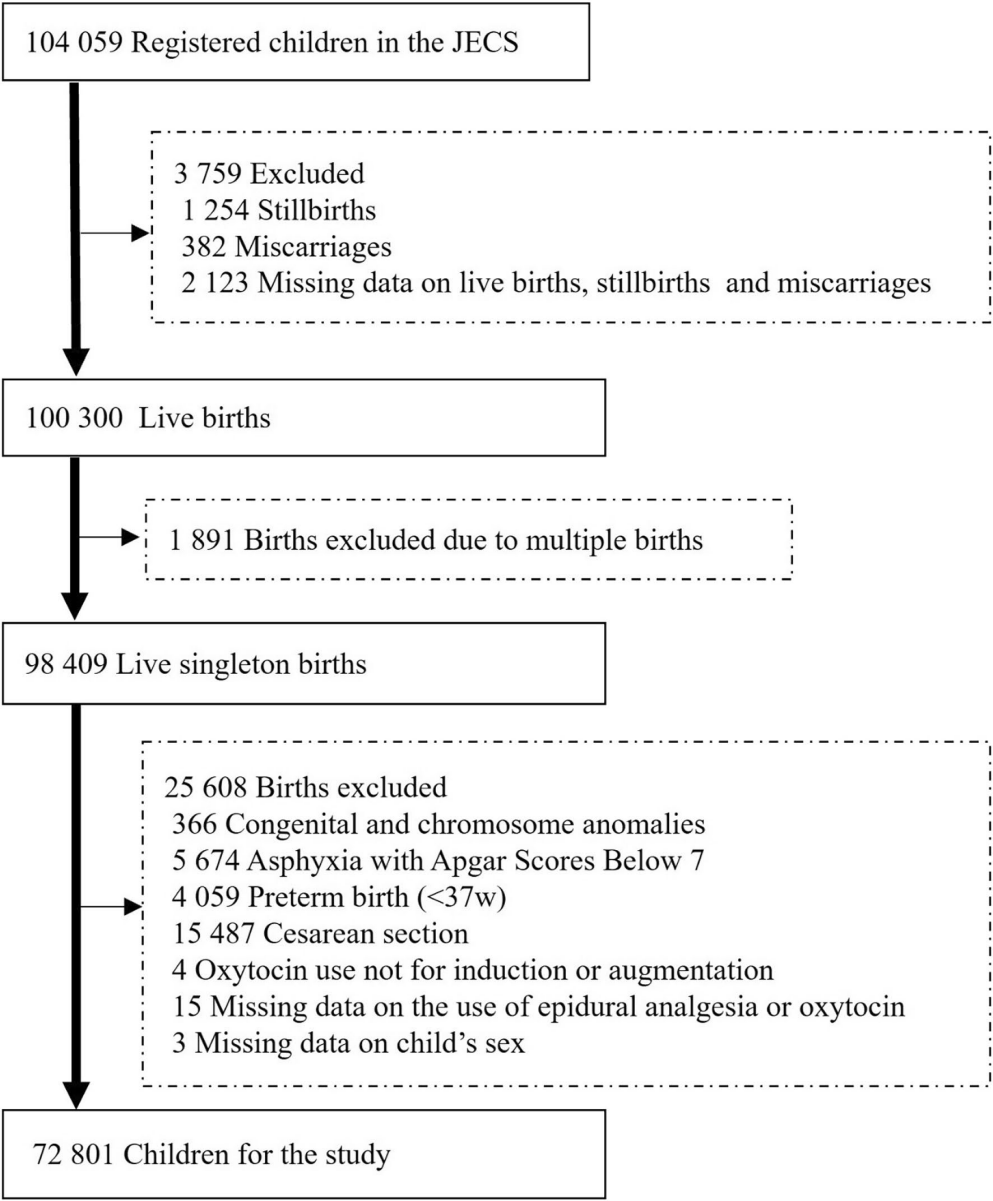
Study enrollment flowchart. *JECS* the Japan Environment and Children’s Study.

After applying the exclusion criteria, 72,801 children were included in the present study (Fig. 1). The characteristics of the participants are summarized in Table 1. The distribution across the four groups was 78.0% in the No exposure group, 19.8% in the OT group, 0.8% in the LEA group, and 1.3% in the LEA-OT group. The LEA and LEA-OT groups exhibited higher maternal age at delivery, educational levels, prevalence of psychiatric disorders, and rates of instrumental delivery compared to the No exposure and OT groups. With respect to neonatal outcomes, the proportion of births prior to 38 weeks was higher, as was the proportion of infants with 5-min Apgar scores below 10 compared to the No exposure and OT groups; however, there were no difference in neonatal transfers (Table 1). Table 2 summarizes ASD and neurodevelopmental milestone outcomes based on the Japanese ASQ-3 domain scores at 3 and 4 years of age. The LEA and LEA-OT groups exhibited a higher prevalence of ASD and a tendency to demonstrate below-cutoff scores in the ASQ-3 domains when compared to the No exposure and OT groups at 3 and 4 years of age (Table 2).

**Table 1:** Characteristics of the participants included in the study.

	Total, no. (%)	No exposure, no. (%)	OT, no. (%)	LEA, no. (%)	LEA-OT, no. (%)
	72 801 (100.0)	56 822 (78.0)	14 441 (19.8)	586 (0.8)	952 (1.3)
Maternal					
Geographic area during the early pregnancy period	72 801 (100.0)	56 816 (78.0)	14 434 (19.8)	592 (0.8)	959 (1.3)
Hokkaido	5 424 (7.5)	4 159 (7.3)	1 178 (8.2)	36 (6.1)	51 (5.3)
Miyagi	6 332 (8.7)	4 963 (8.7)	1 247 (8.6)	75 (12.7)	47 (4.9)
Fukushima	9 738 (13.4)	7 672 (13.5)	1 832 (12.7)	100 (16.9)	134 (14.0)
Chiba	4 055 (5.6)	3 200 (5.6)	768 (5.3)	27 (4.6)	60 (6.3)
Kanagawa	5 023 (6.9)	3 657 (6.4)	1 061 (7.4)	55 (9.3)	250 (26.1)
Koshin	5 410 (7.4)	4 000 (7.0)	1 386 (9.6)	6 (1.0)	18 (1.9)
Toyama	4 196 (5.8)	3 412 (6.0)	699 (4.8)	19 (3.2)	66 (6.9)
Aichi	3 404 (4.7)	2 810 (4.9)	527 (3.7)	23 (3.9)	44 (4.6)
Kyoto	2 833 (3.9)	2 545 (4.5)	228 (1.6)	38 (6.4)	22 (2.3)
Osaka	5 827 (8.0)	4 487 (7.9)	1 253 (8.7)	28 (4.7)	59 (6.2)
Hyogo	3 779 (5.2)	3 087 (5.4)	650 (4.5)	11 (1.9)	31 (3.2)
Tottori	2 317 (3.2)	2 055 (3.6)	258 (1.8)	2 (0.3)	2 (0.2)
Kochi	4 617 (6.3)	3 900 (6.9)	716 (5.0)	1 (0.2)	0 (0.0)
Fukuoka	5 707 (7.8)	3 974 (7.0)	1 529 (10.6)	79 (13.3)	125 (13.0)
South Kyushu/Okinawa	4 139 (5.7)	2 895 (5.1)	1 102 (7.6)	92 (15.5)	50 (5.2)
Maternal age at delivery; years (SD)					
< 25	7 892 (10.8)	6 244 (11.0)	1 548 (10.7)	40 (6.8)	60 (6.3)
25–29	21 098 (29.0)	16 586 (29.2)	4 175 (28.9)	122 (20.6)	215 (22.4)
30–34	25 825 (35.5)	20 297 (35.7)	4 945 (34.3)	238 (40.2)	345 (36.0)
≥ 35	17 980 (24.7)	13 685 (24.1)	3 764 (26.1)	192 (32.4)	339 (35.3)
Missing data	6 (0.0)	4 (0.0)	2 (0.0)	0 (0.0)	0 (0.0)
Gravidity; n					
0	21 975 (30.2)	15 552 (27.4)	5 873 (40.7)	165 (27.9)	385 (40.1)
1	24 480 (33.6)	19 774 (34.8)	4 217 (29.2)	207 (35.0)	282 (29.4)
≥ 2	25 780 (35.4)	21 062 (37.1)	4 221 (29.2)	213 (36.0)	284 (29.6)
Missing data	566 (0.8)	428 (0.8)	123 (0.9)	7 (1.2)	8 (0.8)
Parity					
Nulliparous	28 594 (39.3)	19 984 (35.2)	7 863 (54.5)	237 (40.0)	510 (53.2)
Multiparous	42 510 (58.4)	35 546 (62.6)	6 177 (42.8)	347 (58.6)	440 (45.9)
Missing data	1 697 (2.3)	1 286 (2.3)	394 (2.7)	8 (1.4)	9 (0.9)
BMI before pregnancy; kg/m^2^ (SD)					
< 18.5	12 232 (16.8)	9 841 (17.3)	2 106 (14.6)	118 (19.9)	167 (17.4)
18.5 to < 25	53 724 (73.8)	42 083 (74.1)	10 504 (72.8)	437 (73.8)	700 (73.0)
≥ 25	6 771 (9.3)	4 833 (8.5)	1 810 (12.5)	37 (6.3)	91 (9.5)
Missing data	74 (0.1)	59 (0.1)	14 (0.1)	0 (0.0)	1 (0.1)
Mode of delivery					
Spontaneous delivery	51 570 (70.8)	48 447 (85.3)	2 729 (18.9)	332 (56.1)	62 (6.5)
Induction augmentation	15 956 (21.9)	4 685 (8.2)	10 427 (72.2)	125 (21.1)	719 (75.0)
Vacuum/forceps	5 140 (7.1)	3 563 (6.3)	1 267 (8.8)	132 (22.3)	178 (18.6)
Missing data	135 (0.2)	121 (0.2)	11 (0.1)	3 (0.5)	0 (0.0)
Duration of labor; h					
< 4	18 228 (25.0)	15 002 (26.4)	2 948 (20.4)	79 (13.3)	199 (20.8)
4–8	29 607 (40.7)	23 852 (42.0)	5 145 (35.6)	242 (40.9)	368 (38.4)
> 8	23 086 (31.7)	16 516 (29.1)	5 937 (41.1)	264 (44.6)	369 (38.5)
Missing data	1 880 (2.6)	1 446 (2.5)	404 (2.8)	7 (1.2)	23 (2.4)
Labor induction agents					
Oxytocin					
No	57 408 (78.9)	56 816 (100.0)	0 (0.0)	592 (100.0)	0 (0.0)
Yes	15 393 (21.1)	0 (0.0)	14 434 (100.0)	0 (0.0)	959 (100.0)
Sodium prostaglandin sulfate					
No	71 030 (97.6)	55 548 (97.8)	14 009 (97.1)	552 (93.2)	921 (96.0)
Yes	1 771 (2.4)	1 268 (2.2)	425 (2.9)	40 (6.8)	38 (4.0)
Prostaglandin E₂					
No	68 356 (93.9)	54 655 (96.2)	12 310 (85.3)	549 (92.7)	842 (87.8)
Yes	4 445 (6.1)	2 161 (3.8)	2 124 (14.7)	43 (7.3)	117 (12.2)
Prostaglandin F₂α					
No	71 296 (97.9)	55 828 (98.3)	13 934 (96.5)	584 (98.6)	950 (99.1)
Yes	1 505 (2.1)	988 (1.7)	500 (3.5)	8 (1.4)	9 (0.9)
Gemeprost					
No	72 783 (100.0)	56 803 (100.0)	14 429 (100.0)	592 (100.0)	959 (100.0)
Yes	18 (0.0)	13 (0.0)	5 (0.0)	0 (0.0)	0 (0.0)
Others					
No	71 841 (98.7)	56 308 (99.1)	14 012 (97.1)	583 (98.5)	938 (97.8)
Yes	960 (1.3)	508 (0.9)	422 (2.9)	9 (1.5)	21 (2.2)
Anesthesia for labor pain relief					
Epidural anesthesia					
No	71 263 (97.9)	56 816 (100.0)	14 434 (100.0)	6 (1.0)	7 (0.7)
Yes	1 538 (2.1)	0 (0.0)	0 (0.0)	586 (99.0)	952 (99.3)
Combined spinal–epidural anesthesia					
No	72 788 (100.0)	56 816 (100.0)	14 434 (100.0)	586 (99.0)	952 (99.3)
Yes	13 (0.0)	0 (0.0)	0 (0.0)	6 (1.0)	7 (0.7)
Paracervical block					
No	72 780 (100.0)	56 808 (100.0)	14 421 (99.9)	592 (100.0)	959 (100.0)
Yes	21 (0.0)	8 (0.0)	13 (0.1)	0 (0.0)	0 (0.0)
Infertility treatment					
Yes	5 532 (7.6)	3 791 (6.7)	1 541 (10.7)	60 (10.1)	140 (14.6)
No	66 077 (90.8)	52 098 (91.7)	12 697 (88.0)	516 (87.2)	766 (79.9)
Missing data	1 192 (1.6)	927 (1.6)	196 (1.4)	16 (2.7)	53 (5.5)
Maternal complications					
Hypertension					
No	72 230 (99.2)	56 481 (99.4)	14 215 (98.5)	585 (98.8)	949 (99.0)
Yes	571 (0.8)	335 (0.6)	219 (1.5)	7 (1.2)	10 (1.0)
Diabetes mellitus					
No	72 164 (99.1)	56 411 (99.3)	14 218 (98.5)	586 (99.0)	949 (99.0)
Yes	637 (0.9)	405 (0.7)	216 (1.5)	6 (1.0)	10 (1.0)
Psychiatric disorders					
No	72 246 (99.2)	56 453 (99.4)	14 271 (98.9)	583 (98.5)	939 (97.9)
Yes	555 (0.8)	363 (0.6)	163 (1.1)	9 (1.5)	20 (2.1)
Hypertensive disorders of pregnancy					
No	72 746 (99.9)	56 794 (100.0)	14 402 (99.8)	592 (100.0)	958 (99.9)
Yes	55 (0.1)	22 (0.0)	32 (0.2)	0 (0.0)	1 (0.1)
Gestational diabetes mellitus					
No	71 069 (97.6)	55 634 (97.9)	13 930 (96.5)	579 (97.8)	926 (96.6)
Yes	1 732 (2.4)	1 182 (2.1)	504 (3.5)	13 (2.2)	33 (3.4)
Perinatal complications*					
No	71 926 (98.8)	56 381 (99.2)	14 022 (97.1)	585 (98.8)	938 (97.8)
Yes	875 (1.2)	435 (0.8)	412 (2.9)	7 (1.2)	21 (2.2)
Maternal education level					
≤ High school	25 979 (35.7)	20 293 (35.7)	5 203 (36.0)	197 (33.3)	286 (29.8)
College	29 978 (41.2)	23 288 (41.0)	6 074 (42.1)	223 (37.7)	393 (41.0)
≥ Bachelor's degree	15 454 (21.2)	12 123 (21.3)	2 909 (20.2)	161 (27.2)	261 (27.2)
Missing data	1 390 (1.9)	1 112 (2.0)	248 (1.7)	11 (1.9)	19 (2.0)
Marital status					
Married	68 456 (94.0)	53 486 (94.1)	13 541 (93.8)	553 (93.4)	876 (91.3)
Never married	2 616 (3.6)	1 978 (3.5)	601 (4.2)	15 (2.5)	22 (2.3)
Divorced or widowed	553 (0.8)	440 (0.8)	99 (0.7)	7 (1.2)	7 (0.7)
Missing data	1 176 (1.6)	912 (1.6)	193 (1.3)	17 (2.9)	54 (5.6)
Maternal smoking status					
Never smoked	41 788 (57.4)	32 794 (57.7)	8 159 (56.5)	326 (55.1)	509 (53.1)
Quit smoking before pregnancy	16 803 (23.1)	13 128 (23.1)	3 283 (22.7)	151 (25.5)	241 (25.1)
Smoked during pregnancy	12 813 (17.6)	9 814 (17.3)	2 754 (19.1)	95 (16.0)	150 (15.6)
Missing data	1 397 (1.9)	1 080 (1.9)	238 (1.6)	20 (3.4)	59 (6.2)
Alcohol consumption					
Never drank	24 867 (34.2)	19 469 (34.3)	4 897 (33.9)	196 (33.1)	305 (31.8)
Ex-drinker	39 641 (54.5)	30 741 (54.1)	8 062 (55.9)	319 (53.9)	519 (54.1)
Current drinker	7 112 (9.8)	5 671 (10.0)	1 300 (9.0)	61 (10.3)	80 (8.3)
Missing data	1 181 (1.6)	935 (1.6)	175 (1.2)	16 (2.7)	55 (5.7)
Annual household income; million JPY					
< 4	26 943 (37.0)	21 196 (37.3)	5 340 (37.0)	173 (29.2)	234 (24.4)
4–8	32 571 (44.7)	25 438 (44.8)	6 371 (44.1)	277 (46.8)	485 (50.6)
≥ 8	7 149 (9.8)	5 422 (9.5)	1 474 (10.2)	90 (15.2)	163 (17.0)
Missing data	6 138 (8.4)	4 760 (8.4)	1 249 (8.7)	52 (8.8)	77 (8.0)
Household					
Paternal education level					
≤ High school	31 458 (43.2)	24 656 (43.4)	6 274 (43.5)	218 (36.8)	310 (32.3)
College	16 059 (22.1)	12 469 (21.9)	3 256 (22.6)	130 (22.0)	204 (21.3)
≥ Bachelor’s degree	23 425 (32.2)	18 211 (32.1)	4 561 (31.6)	232 (39.2)	421 (43.9)
Missing data	1 859 (2.6)	1 480 (2.6)	343 (2.4)	12 (2.0)	24 (2.5)
Paternal smoking status					
Never smoked	19 075 (26.2)	14 893 (26.2)	3 735 (25.9)	173 (29.2)	274 (28.6)
Quit smoking before pregnancy	16 353 (22.5)	12 841 (22.6)	3 130 (21.7)	146 (24.7)	236 (24.6)
Smoked during pregnancy	34 990 (48.1)	27 240 (47.9)	7 119 (49.3)	248 (41.9)	383 (39.9)
Missing data	2 383 (3.3)	1 842 (3.2)	450 (3.1)	25 (4.2)	66 (6.9)
Offspring					
Sex					
Male	37 072 (50.9)	29 025 (51.1)	7 266 (50.3)	274 (46.3)	507 (52.9)
Female	35 729 (49.1)	27 791 (48.9)	7 168 (49.7)	318 (53.7)	452 (47.1)
Gestational age at birth; week					
< 38	18 142 (24.9)	14 314 (25.2)	3 256 (22.6)	172 (29.1)	400 (41.7)
39–40	47 291 (65.0)	38 020 (66.9)	8 429 (58.4)	365 (61.7)	477 (49.7)
> 41	7 368 (10.1)	4 482 (7.9)	2 749 (19.0)	55 (9.3)	82 (8.6)
Body weight; g					
< 2500	3 270 (4.5)	2 572 (4.5)	620 (4.3)	31 (5.2)	47 (4.9)
2500–4000	68 899 (94.6)	53 819 (94.7)	13 613 (94.3)	558 (94.3)	909 (94.8)
> 4000	623 (0.9)	417 (0.7)	200 (1.4)	3 (0.5)	3 (0.3)
Missing data	9 (0.0)	8 (0.0)	1 (0.0)	0 (0.0)	0 (0.0)
Apgar scores at 5 min					
7–9	37 403 (51.4)	28 409 (50.0)	7 875 (54.6)	427 (72.1)	692 (72.2)
10	35 398 (48.6)	28 407 (50.0)	6 559 (45.4)	165 (27.9)	267 (27.8)
Neonatal transport					
No	69 652 (95.7)	54 564 (96.0)	13 597 (94.2)	573 (96.8)	918 (95.7)
Yes	2 216 (3.0)	1 508 (2.7)	658 (4.6)	16 (2.7)	34 (3.5)
Missing data	933 (1.3)	744 (1.3)	179 (1.2)	3 (0.5)	7 (0.7)
Feeding at 1 month of age					
Breast milk only or mixed feeding	70 443 (96.8)	55 003 (96.8)	13 956 (96.7)	571 (96.5)	913 (95.2)
Formula only	904 (1.2)	659 (1.2)	209 (1.4)	11 (1.9)	25 (2.6)
Missing data	1 454 (2.0)	1 154 (2.0)	269 (1.9)	10 (1.7)	21 (2.2)
Sibling cohabitation					
No	31 859 (43.8)	22 855 (40.2)	8 224 (57.0)	256 (43.2)	524 (54.6)
Yes	39 677 (54.5)	32 957 (58.0)	5 978 (41.4)	327 (55.2)	415 (43.3)
Missing data	1 265 (1.7)	1 004 (1.8)	232 (1.6)	9 (1.5)	20 (2.1)
Nursery attendance at 1 year of age					
Yes	47 963 (65.9)	37 386 (65.8)	9 524 (66.0)	402 (67.9)	651 (67.9)
No	17 814 (24.5)	13 949 (24.6)	3 518 (24.4)	132 (22.3)	215 (22.4)
Missing data	7 024 (9.6)	5 481 (9.6)	1 392 (9.6)	58 (9.8)	93 (9.7)

Data represent number (%). *No exposure*, no exposure to either labor epidural analgesia or oxytocin administration during labor; *OT*, exposure to oxytocin administration without labor epidural analgesia during labor; *LEA*, labor epidural analgesia without oxytocin administration during labor; *LEA–OT*, both labor epidural analgesia and oxytocin administration during labor; *SD*, standard deviation; *BMI*, body mass index (calculated as weight in kilograms divided by height in meters squared); *JPY*, Japanese yen. *Fetal distress, non-reassuring fetal status, prolonged or arrested labor, preterm rupture of membranes, intrauterine infection, chorioamnionitis and post-term pregnancy.

**Table 2:** Descriptions of the ASD and Below the cut-off of ASQ-3 domains at 3 and 4 years of age.

Descriptions	At 3 years of age no. (%)				At 4 years of age no. (%)			
ASD	No exposure	OT	LEA	LEA-OT	No exposure	OT	LEA	LEA-OT
No	46 900 (99.6)	11 875 (99.6)	496 (99.4)	789 (98.7)	44 163 (99.1)	11 182 (98.8)	452 (97.4)	726 (97.8)
Yes	188 (0.4)	52 (0.4)	3 (0.6)	10 (1.3)	423 (0.9)	134 (1.2)	12 (2.6)	16 (2.2)

Below the cut-off of ASQ-3 domains	No exposure	OT	LEA	LEA-OT	No exposure	OT	LEA	LEA-OT
Communication								
No	43 475 (96.4)	11 049 (96.3)	465 (95.9)	731 (94.1)	40 532 (95.7)	10 323 (95.7)	430 (95.8)	677 (94.3)
Yes	1 620 (3.6)	426 (3.7)	20 (4.1)	46 (5.9)	1 809 (4.3)	465 (4.3)	19 (4.2)	41 (5.7)
Gross motor								
No	43 382 (96.1)	11 004 (95.8)	468 (96.5)	732 (94.0)	40 296 (94.8)	10 225 (94.4)	430 (94.9)	669 (93.3)
Yes	1 768 (3.9)	484 (4.2)	17 (3.5)	47 (6.0)	2 214 (5.2)	601 (5.6)	23 (5.1)	48 (6.7)
Fine motor								
No	41 853 (93.1)	10 593 (92.5)	452 (93.2)	711 (91.5)	39 870 (93.8)	10 133 (93.7)	425 (94.2)	654 (90.8)
Yes	3 113 (6.9)	863 (7.5)	33 (6.8)	66 (8.5)	2 649 (6.2)	684 (6.3)	26 (5.8)	66 (9.2)
Problem solving								
No	41 670 (93.2)	10 585 (92.9)	444 (91.7)	708 (91.2)	41 025 (96.7)	10 447 (96.7)	438 (97.1)	694 (96.4)
Yes	3 062 (6.8)	803 (7.1)	40 (8.3)	68 (8.8)	1 415 (3.3)	359 (3.3)	13 (2.9)	26 (3.6)
Personal-social								
No	43 723 (97.1)	11 132 (97.1)	472 (97.5)	743 (95.5)	40 352 (94.8)	10 287 (94.9)	428 (94.5)	666 (92.8)
Yes	1 328 (2.9)	328 (2.9)	12 (2.5)	35 (4.5)	2 207 (5.2)	550 (5.1)	25 (5.5)	52 (7.2)

*ASD* autism spectrum disorder; *ASQ-3* ages and stages questionnaires, Third Edition; No exposure, no exposure to either labor epidural analgesia or oxytocin administration during labor; *OT* exposure to oxytocin administration without labor epidural analgesia during labor; *LEA* labor epidural analgesia without oxytocin administration during labor; *LEA–OT* both labor epidural analgesia and oxytocin administration during labor.

### Association of LEA and OT exposure with offspring ASD and neurodevelopmental milestones at 3 and 4 years of age

The LEA-OT and LEA groups exhibited a statistically significant higher adjusted odds ratio (aOR) for ASD after FDR correction (aOR 2.35 [95% CI 1.44–3.83] and aOR 2.41 [95% CI 1.31–4.45], respectively) (Table 3). Only the LEA-OT group showed increased aOR for developmental delay in communication and social personal skills in the ASQ-3 domains (aOR 1.40 [95% CI 1.07–1.82] and aOR 1.30 [95% CI 1.00–1.69], respectively), though none remained significant after FDR correction (Table 3). The interaction terms between LEA and OT were not statistically significant for either ASD or neurodevelopmental delay.

**Table 3:** Association of of labor epidural analgesia and/or oxytocin exposure with offspring ASD and neurodevelopmental milestones^a^.

	No. (%)	ASD, aOR [95% CI]	Below the cut off in ASQ-3 domains, aOR [95% CI]				
	72 801 (100.0)		Communication	Gross motor	Fine motor	Problem solving	Social personal
No exposure	56 822 (78.1)	1 [Reference]	1 [Reference]	1 [Reference]	1 [Reference]	1 [Reference]	1 [Reference]
OT	14 441 (19.8)	1.05 [0.82—1.35]	0.99 [0.88—1.12]	1.05 [0.82—1.35]	1.00 [0.92—1.08]	0.96 [0.87—1.06]	0.93 [0.83—1.04]
LEA	586 (0.81)	2.41^b^ [1.31—4.45]	1.15 [0.80—1.67]	0.89 [0.63—1.26]	1.01 [0.75—1.36]	1.13 [0.84—1.52]	1.00 [0.70—1.45]
LEA-OT	952 (1.31)	2.35 ^b^ [1.44—3.83]	1.40 [1.07—1.82]	1.16 [0.92—1.48]	1.21 [0.99—1.50]	1.14 [0.90—1.44]	1.30 [1.00—1.69]

^a^Multiple imputation generalized estimating equations. ^b^*q* < 0.05. Adjusted for geographic area during the early pregnancy period, maternal age at birth, gravidity, parity, body mass index before pregnancy, mode of delivery, duration of labor, infertility treatment, hypertension, diabetes mellitus, psychiatric disorders, hypertensive disorders of pregnancy, gestational diabetes mellitus, perinatal complications, maternal education level, marital status, maternal smoking status, maternal alcohol consumption, annual household income, paternal education level, paternal smoking status, children’s sex, gestational age at birth, birth weight, 5-min Apgar score, and nursery attendance at 1 year. *ASD* autism spectrum disorder; *ASQ-3* the Ages and Stages Questionnaires, Third Edition; *aOR* adjusted odds ratio; *No exposure* no exposure to either labor epidural analgesia or oxytocin administration during labor; *OT* exposure to oxytocin administration without labor epidural analgesia during labor; *LEA* labor epidural analgesia without oxytocin administration during labor; *LEA–OT* both labor epidural analgesia and oxytocin administration during labor.

### Sensitivity analyses

#### E-value analysis

To assess the robustness of our observed associations to potential unmeasured confounding, we calculated the E-values for each association. For the LEA-OT versus No exposure groups with ASD, the E-values of 4.16 for the point estimate and 2.39 for the lower 95% CI limit, suggesting that this association is only moderately robust to unmeasured confounding (eTable 2 in the Supplement). Similarly, for the LEA versus No exposure groups with ASD, the E-values of 4.29 for the point estimate and 2.05 for the lower 95% CI limit indicated a comparable level of robustness (eTable 2 in the Supplement). For the LEA-OT versus No exposure groups with the E-values of ASQ‑3 communication and social-personal skills domains also showed associations that appeared week to moderately robust to potential unmeasured confounding, although to a lesser extent than that observed for ASD (eTable 2 in the Supplement).

#### Inverse probability weighting (IPW) analysis

The LEA-OT and LEA groups showed higher aOR for ASD (aOR 2.21 [95% CI 1.21–4.03] and aOR 2.59 [95% CI 1.30–5.20], respectively), but significance was lost after FDR correction (Table 4). The OT group showed no associations with ASD (Table 4). None of the groups showed increased aOR and reached statistical significance for developmental delays in any of the ASQ-3 domains (Table 4).

**Table 4:** Sensitivity analysis using inverse probability weighting in generalized estimating equation models.

	No. (%)	ASD, aOR [95% CI]	Below the cut off in ASQ-3 domains, aOR [95% CI]				
	61 970 (100.0)		Communication	Gross motor	Fine motor	Problem solving	Social personal
No exposure	48 372 (78.1)	1 [Reference]	1 [Reference]	1 [Reference]	1 [Reference]	1 [Reference]	1 [Reference]
OT	12 272 (19.8)	1.03 [0.77–1.39]	0.95 [0.82–1.09]	1.00 [0.88–1.12]	1.01 [0.91–1.12]	0.95 [0.85–1.07]	0.98 [0.86–1.12]
LEA	510 (0.8)	2.59 [1.30–5.20]	1.12 [0.70–1.80]	0.95 [0.64–1.42]	1.09 [0.77–1.56]	1.19 [0.83–1.70]	1.12 [0.74–1.69]
LEA-OT	816 (1.3)	2.21 [1.21–4.03]	1.17 [0.82–1.66]	0.99 [0.73–1.34]	1.18 [0.91–1.55]	1.04 [0.77–1.41]	1.29 [0.95–1.75]

Adjusted for geographic area during the early pregnancy period, maternal age at birth, gravidity, parity, body mass index before pregnancy, mode of delivery, duration of labor, infertility treatment, hypertension, diabetes mellitus, psychiatric disorders, hypertensive disorders of pregnancy, gestational diabetes mellitus, perinatal complications, maternal education level, marital status, maternal smoking status, maternal alcohol consumption, annual household income, paternal education level, paternal smoking status, children's sex, gestational age at birth, birth weight, 5-min Apgar score, and nursery attendance at 1 year. *ASD* autism spectrum disorder; *ASQ-3* the Ages and Stages Questionnaires, Third Edition; *aOR* adjusted odds ratio; *No exposure* no exposure to either labor epidural analgesia or oxytocin administration during labor; *OT* exposure to oxytocin administration without labor epidural analgesia during labor; *LEA* labor epidural analgesia without oxytocin administration during labor; *LEA–OT* both labor epidural analgesia and oxytocin administration during labor. Analyses were based on outcome-complete cases, which varied across outcomes due to differential missing patterns, while the reported total case reflects the full cohort used for inverse probability weight estimation.

#### Complete-case analysis

The LEA-OT and LEA groups showed higher aOR for ASD (aOR 2.27 [95% CI 1.25–4.13] and aOR 2.15 [95% CI 1.01–4.59], respectively), but significance was lost after FDR correction (eTable 3 in the Supplement). The OT group showed no significance (eTable 3 in the Supplement). None of the groups showed increased aOR in any of the ASQ-3 domains (eTable 3 in the Supplement).

#### Sex-stratified analysis

Among males, the LEA-OT and LEA groups showed higher odds ratio for ASD (aOR, 2.67 [95% CI 1.31–5.43] and aOR 2.30 [95% CI 1.03–5.13], respectively), but significance was lost after FDR correction. The OT group showed no association with ASD (eTable 4 in the Supplement). For the ASQ-3 domains, only the LEA-OT group demonstrated elevated odds ratios for developmental delays in social personal skills (aOR 1.44 [95% CI 1.00–2.06]), though it did not remain significant after FDR correction (eTable 4 in the Supplement). Among females, none of the groups showed increased aOR for ASD and developmental delays in any of the ASQ-3 domains (eTable 4 in the Supplement).

#### Independent Correlation Structure analysis

Only the LEA-OT group demonstrated statistically significant higher aOR for ASD after FDR correction (aOR 2.62 [95% CI 1.40–4.91]), whereas the LEA and OT groups did not show associations (eTable 5 in the Supplement). None of the groups showed increased aOR for developmental delays in any of the ASQ-3 domains (eTable 5 in the Supplement).

#### Additionally adjusted analysis

Only the LEA-OT group showed higher odds ratio for ASD (aOR 2.53 [95% CI 1.32–4.83]), although it did not remain significant after FDR correction (eTable 6 in the Supplement). None of the groups showed increased aOR for developmental delays in any of the ASQ-3 domains (eTable 6 in the Supplement).

#### Restricted to oxytocin as the sole labor induction agent analysis

Only the LEA-OT group demonstrated higher odds ratio for ASD (aOR 2.62 [95% CI 1.38–4.97]), though this was not statistically significant after FDR correction (eTable 7 in the Supplement). None of the groups showed increased aOR for developmental delays in any of the ASQ-3 domains (eTable 7 in the Supplement).

## Discussion

Main analyses showed statistically significantly higher adjusted odds ratios for ASD and both LEA-OT and LEA groups after FDR correction, whereas the OT group showed no association. Only the LEA-OT group showed association with ASQ-3 domains of social personal skills, though this did not survive FDR correction. For the association between LEA or LEA-OT and ASD, E-values (limit of 95% CI) of approximately 2.0 in both groups indicated that these are only moderately robust to unmeasured confounding and therefore should be interpreted with particular caution, even though the higher E-values (point estimate) of approximately 4.0 suggested that explaining the point estimates would require at most moderate associations of unmeasured confounders^38^. IPW and complete-case analysis found associations between ASD and both LEA-OT and LEA groups that became non-significant after FDR correction. Sex‑stratified analyses revealed that only males showed higher odds ratios for ASD in the LEA‑OT and LEA groups, and for developmental delays only in the LEA‑OT group, specifically in the social–personal skills domain of ASQ‑3; however, these associations did not remain significant after FDR correction. Alternative correlation structures analysis showed that only the LEA-OT group showed elevated odds and statistically significance after FDR correction. Additionally adjusted analysis showed associations between ASD and LEA-OT group, but this did not reach significance after FDR correction. Restricted to oxytocin as the solo labor induction agent analysis showed associations between ASD and LEA-OT group, but this did not reach significance after FDR correction. Across all analyses with ASQ-3 domains, no significant associations of any groups were observed.

Although main and sensitivity analytical approaches demonstrated consistency, the discrepancies observed between the results of missing imputed and complete-case analysis can be attributed to the underlying missing data mechanism operating under the missing at random (MAR) rather than missing completely at random (MCAR) assumption^39^. Multiple imputation methodologies have consistently exhibited superior bias reduction properties under MAR conditions compared with complete-case approaches, which generate biased estimates when missingness is systematically related to observed characteristics^40^. Under MAR conditions, complete-case analysis generates biased estimates while multiple imputation generally produces estimates with smaller bias^39^. Sex differences have been reported in both ASD prevalence and neurodevelopment^1^. In the sex-stratified analysis, increased odds ratios for ASD and developmental delay in the ASQ-3 domains were observed only in the male group within the LEA-OT group. This may reflect limited statistical power to detect sex-specific effects, attributable to insufficient sample sizes in sex-stratified analyses and greater clinical heterogeneity among female participants^41^.

ELA for labor pain relief in Japan was lower than in other high‑income countries, with its use at only 2–3% in 2009 and 2% in the JECS, which was launched in 2011^10,17^. This root cause is the limited number of qualified anesthesiologists and restricted availability at the private obstetric facilities that handle approximately half of all the deliveries^42^. In this study, LEA alone showed associations in some analyses but no consistently significant associations overall. A meta-analysis examining associations of LEA with ASD in offspring across 16 studies reported that association may be largely attributable to confounding factors, as it disappeared in sibling analysis^43^. While JECS-based studies have demonstrated significant associations between LEA and ASD and neurodevelopmental delay with ASQ-3 up to 3 years of age^10,14^, some associations between LEA and neurodevelopmental delays in ASQ-3 domains were observed in the present study, but they did not reach statistical significance. This may reflect the separation of LEA-OT and LEA exposures, the evaluation of six outcomes, including ASD, and five ASQ-3 domains across four exposure groups with FDR correction for multiple comparisons, as well as the longitudinal modeling of repeated outcomes up to 4 years of age.

The OT group also showed no significant associations in the present study. A meta-analysis reported a small pooled risk of OT for ASD, but the evidence remains insufficient to establish causality^44^. A large cohort study demonstrated no association between OT and offspring ASD but showed an association with LEA-OT^21^, consistent with the present study findings. Furthermore, an ASQ-3 report from JECS observed no association between intrapartum OT administration and delayed offspring neurodevelopment up to 3 years of age^26^, also consistent with the present study findings.

To propose a mechanism specifically linked to ASD disorder rather than general neurodevelopmental disorders, the combined use of LEA and synthetic OT must selectively target neural circuits mediating social communication and repetitive behaviors. Due to their lipophilic properties, anesthetics for LEA such as bupivacaine and fentanyl cross the placenta and enter fetal circulation^45,46^. In animal studies, anesthetic μ-opioid receptor agonists suppress OT-producing neurons within the hypothalamus, which may inhibit OT release from the posterior pituitary and direct OT projections from the paraventricular nucleus to the neonatal hippocampus, where OT receptors mediate discrimination of social stimuli^47–49^. However, the long-term neurodevelopmental effects remain unclear. Synthetic OT is likely to cross the placenta^50^, and animal studies have demonstrated that perinatal OT exposure can cause brain damage, cortical volume loss and altered functional connectivity in male offspring^51,52^. In addition, an in vitro study suggested that exposure to synthetic OT may downregulate OT receptor expression in the neural progenitor cell^53^. These alterations may disrupt OT-dependent neurogenesis and circuit formation in the dentate gyrus of the hippocampus via OT receptors in CA3 pyramidal neurons^49^. Consequently, this could impair social recognition and discrimination of social stimuli^48^, contributing to the social communication deficits characteristic of ASD. Although the interaction terms were not statistically significant, the associations with ASD were observed only among offspring exposed to both LEA and OT, indicating that potential pharmacological interactions cannot be excluded and warrant further investigation into their combined effects and underlying mechanisms.

Several strengths of this study are noteworthy. First, this is the first study to comprehensively assess the neurological outcomes of offspring exposed to intrapartum LEA and OT using a nationwide birth cohort in Japan. Second, most previous studies have evaluated exposure to either LEA or OT alone, whereas our study specifically examined combined exposure to LEA and OT and its association with offspring ASD and neurodevelopment, thereby providing novel insights beyond these earlier reports^9–14,21–23,26,30,31^. Third, the study’s longitudinal design, which included participants representative of the Japanese population^54^, enhances the generalizability and credibility of our results.

Nonetheless, this study has some limitations. First, although the participants were recruited from the general Japanese population, selection bias is possible. Second, while the JECS is a longitudinal birth cohort study in Japan^54^, the lack of randomization, data limitations, and potential observer bias cannot be ruled out. Additionally, OT and LEA are used according to clinical needs, making it difficult to conduct randomized controlled trials. Third, the questionnaire, which was based on the developmental scores reported by guardians, may lead to overestimation or other misclassifications. Fourth, because data on LEA and OT during labor were obtained from binary data (used or not) recorded by physicians immediately after birth, details such as dose or time duration were not available. Finally, residual confounding may have existed. Although E-value analysis was performed in this study, the demonstrated associations showed only moderate robustness to unmeasured confounders, indicating that residual confounding cannot be excluded. Maternal educational level and age at delivery were adjusted for in the analysis; nonetheless, mothers in the LEA–OT group tended to have higher educational levels and were older at delivery, which may have influenced the likelihood of choosing LEA and has been reported to increase the probability of accessing medical facilities capable of diagnosing ASD^55^. In addition to the confounders, particularly important unadjusted factors include paternal age and geographic access to specialized medical facilities diagnosing ASD^7,56^. However, the amount of available data on the latter factors were not included in the dataset and could not be incorporated as covariates. Consequently, it cannot be ruled out that these unmeasured factors introduced bias into the observed associations, requiring caution in interpreting the study results. Therefore, the observed associations should be interpreted conservatively, and inferences of causality require careful consideration.

In conclusion, this is the first large-scale longitudinal birth cohort study to investigate the association between intrapartum LEA and OT and offspring ASD and neurodevelopmental outcomes through 4 years of age in Japan. Intrapartum LEA-OT exposure was consistently associated with ASD, especially in males, while neurodevelopmental delays were not. Although statistical significance was observed, these findings should not be interpreted as establishing causality due to unmeasured confounding factors, such as geographic accessibility to specialized medical facilities, along with limited external validity, and require cautious interpretation and replication in future studies.

## Methods

### Study population and data sources

This study analyzed data from the Japan Environment and Children’s Study (JECS), a comprehensive nationwide multicenter birth cohort study funded by the Ministry of the Environment of Japan. The JECS protocol adhered to strict ethical standards in accordance with the Declaration of Helsinki and the Japanese Ethical Guidelines for Medical and Biological Research Involving Human Subjects. The JECS registered 104,059 fetal records from January 2011 to March 2014 at 15 regional centers in Japan^54,57^. This study was reviewed and approved by the Ministry of the Environment's Institutional Review Board on the Epidemiological Studies and the Ethics Committees of all participating institutions (ethical approval number: 100910001). Written informed consent was obtained from all the participants. The present study analyzed the “jecs-ta-20190930” and “jecs-qa-20210401” datasets. The characteristics of all JECS participants closely matched those reported in the 2013 Japanese Vital Statistics Survey, suggesting that the study sample was representative of a broader population^58^. This study follows the Strengthening the Reporting of Observational Studies in Epidemiology (STROBE) reporting guideline^59^. A total of 72,801 participants were included in the present study from 104,059 individuals who were enrolled in the JECS. We restricted to the participants with live-born singleton births. The exclusion criteria were congenital disease or chromosomal abnormalities; preterm birth (< 37 weeks); low Apgar score at 5 min (≤ 6); children born via cesarean section; missing data for children’s sex or exposure to OT or LEA during labor; and exposure to OT to reduce bleeding after labor (Fig. 1).

### Exposures

LEA and OT were the primary exposure variables. Presence or absence of these exposures for labor induction was assessed from the medical record transcripts. Other labor induction agents, in addition to OT, were categorized in the OT group. Similarly, LEA use in combination with spinal anesthesia or other anesthetic methods was categorized in the LEA group.

### Autism and neurodevelopmental assessment

ASD was assessed using a questionnaire administered to the guardians of participating children aged 3 and 4 years. The guardians were asked the following question beginning when their child was 3 years old: “Has your child been diagnosed by a doctor with an ASD (e.g. autism, pervasive developmental disorder, Asperger’s syndrome)?” Children with a “yes” response were classified as having ASD, and those with a “no” response as not having ASD.

The Ages and Stages Questionnaires, Third Edition (ASQ-3) was used for neurodevelopmental screening, which includes 21 guardian questionnaires across communication, gross motor, fine motor, problem-solving, and Personal-social characteristic domains for children aged 1–66 months^60^. The results from the 36- and 48-month ASQ-3 questionnaires were analyzed. The Japanese translation of the ASQ-3 has been validated ASQ-3 cut off scores for Japanese children^61^. Details of the ASD and neurodevelopmental assessments are presented in the eMethods and eTable 1.

### Variables

We considered potential variables based on relevant literature and clinical plausibility, using established categorization criteria where available ^3,4,6–8,62–64^ Maternal characteristics included the geographic area during the early pregnancy period (Hokkaido, Miyagi, Fukushima, Chiba, Kanagawa, Koshin, Toyama, Aichi, Kyoto, Osaka, Hyogo, Tottori, Kochi, Fukuoka, and South Kyushu/Okinawa), age at birth (< 25, 25–29, 30–34, ≥ 35 years), gravidity (0, 1, and ≥ 2), parity (nulliparous or multiparous), body mass index before pregnancy (< 18.5, 18.5 to < 25, and ≥ 25 kg/m^2^), mode of delivery (spontaneous delivery, induction augmentation, vacuum/ forceps), duration of labor (< 4, 4–8, and > 8 h), labor induction agents (oxytocin, sodium prostaglandin sulfate, prostaglandin E₂, prostaglandin F₂, gemeprost, and others), anesthesia for labor pain relief (LEA, combined spinal–LEA, and paracervical block), infertility treatment, hypertension, diabetes mellitus, psychiatric disorders, hypertensive disorders of pregnancy, gestational diabetes mellitus, perinatal complications (fetal distress, non-reassuring fetal status, prolonged or arrested labor, preterm rupture of membranes, intrauterine infection, chorioamnionitis, and post-term pregnancy), education level (≤ high school, college, and ≥ bachelor’s degree), marital status (married, never married, and divorced or widowed), smoking status (never smoked, quit smoking before pregnancy, and smoked during pregnancy), alcohol consumption (never drank, ex-drinker, and current drinker), and annual household income (< 4, 4–8, and ≥ 8 million JPY). Household characteristics included paternal education level (≤ high school, college, and ≥ bachelor's degree) and smoking status (never smoked, quit smoking before pregnancy, and smoked during pregnancy). Child-related variables included sex, gestational age at birth (< 38, 39–40, and 41 > week), birth weight (< 2500, 2500–4000, and > 4000 g), 5-min Apgar score (7–9 and 10), neonatal transport, feeding method at 1 month of age (breast milk only, mixed feeding and formula only), sibling cohabitation status, and nursery attendance at 1 year.

### Statistical analyses

We applied a stratified analysis to investigate the potential biological and synergistic effects of the combined use of LEA and OT based on the following groups: No exposure, OT, LEA and LEA-OT^65^. Little’s MCAR test suggested data were not MCAR (*P* < 0.05); thus, we proceeded under the assumption of MAR^66^. Longitudinal associations between intrapartum exposure to LEA and OT and offspring ASD and neurodevelopmental milestones were assessed as the main analysis using multiple imputation- generalized estimating equations (MI-GEE) to appropriately handle missing data. Multiple imputation using chained equations was performed to generate 20 imputed datasets that included all analysis variables, including ASD and ASQ-3 outcomes with missing values^40^. Generalized estimating equations (GEE) with a binomial distribution, logit link, and an exchangeable correlation structure was then applied to each imputed dataset^67^. Robust standard errors (Huber–White sandwich estimator) were used to estimate odds ratios and 95% confidence intervals (CI). The models included geographic area during the early pregnancy period, maternal age at birth, gravidity, parity, body mass index before pregnancy, mode of delivery, duration of labor, infertility treatment, hypertension, diabetes mellitus, psychiatric disorders, hypertensive disorders of pregnancy, gestational diabetes mellitus, perinatal complications, maternal education level, marital status, maternal smoking status, maternal alcohol consumption, annual household income, paternal education level, paternal smoking status, children’s sex, gestational age at birth, birth weight, 5-min Apgar score, and nursery attendance at 1 year as covariates. To assess the robustness of the main results, we conducted several sensitivity analyses. To assess the robustness of the main results, we conducted several sensitivity analyses. To evaluate sensitivity to unmeasured confounding, we calculated the E-values, which quantify the minimum strength of association that an unmeasured confounder would need to have with both the exposure and outcome to fully explain the observed effect^68^. The E-value for the point estimate quantifies the minimum strength of unmeasured confounding needed to explain away the observed association, whereas the E-value for the 95% CI limit quantifies the minimum strength needed to shift the confidence interval to include the null^68^. IPW-GEE was conducted as a sensitivity analysis to address potentially informative loss to follow-up (missing outcomes) by weighting each observation with the inverse of the estimated probability of remaining under observation at each time point, conditional on prognostic baseline and perinatal covariates^69^. Complete-case GEE analyses were conducted, including only participants with complete data on all variables included in the analysis. Given the established sex differences in neurodevelopment, we further conducted sex-stratified GEE analyses to examine potential effect modification by sex. Subsequently, we performed GEE analysis with an independent correlation structure to validate the main findings with an exchangeable correlation structure and additional GEE models with further adjustments for neonatal transport, feeding at 1 month, and sibling cohabitation were performed. Finally, to eliminate the influence of agents for induction with different pharmacological mechanisms, we conducted GEE models restricted to participants exposed to oxytocin as the sole labor induction agent.

Raw P-values from all hypothesis tests were adjusted for multiple comparisons using the Benjamini–Hochberg method, and results with False Discovery Rate (FDR)-adjusted *P*-values (q-values) < 0.05 were considered statistically significant^70^. To investigate the synergistic effect of the combined use of LEA and OT, we also assessed the interaction terms of ASD and neurodevelopmental delay, with *P* < 0.05 considered statistically significant.

All statistical analyses were performed using StataNow, version 18.5 (StataCorp LLC, College Station, TX, USA).

## Supplementary Information

Supplementary Information.

## Data Availability

Data are unsuitable for public deposition due to ethical restrictions and the legal framework of Japan. It is prohibited by the Act on the Protection of Personal Information (Act No. 57 of 30 May 2003, amended on 9 September 2015) to publicly deposit the data containing personal information. Ethical Guidelines for Medical and Health Research Involving Human Subjects enforced by the Japan Ministry of Education, Culture, Sports, Science and Technology and the Ministry of Health, Labour and Welfare also restricts the open sharing of epidemiologic data. All inquiries about access to data should be sent to: jecs-en@nies.go.jp. The person responsible for handling enquiries sent to this e-mail address is Dr Shoji F. Nakayama, JECS Program Office, National Institute for Environmental Studies.
