# Supplementary material for: Association of intrapartum epidural analgesia and oxytocin exposure with offspring autism and neurodevelopment from the Japan Environment and Children’s Study
Source: Sci Rep. 2026 May 18;16:24417. doi: 10.1038/s41598-026-46497-8 (PMC13447853; doi:10.1038/s41598-026-46497-8)
Supplement: Supplementary file 1 [file 41598_2026_46497_MOESM1_ESM.docx]

**Supplementary Information**

**eMethods. Autism and Neurodevelopmental Assessment**

**eTable 1. Neurodevelopmental Milestones and Validated Cutoff Values in ASQ-3 Domains**

**eTable 2. E-value Sensitivity Analysis for the Association of Epidural Analgesia and Oxytocin Exposure with Offspring ASD and Neurodevelopmental Milestones**

**eTable 3. Sensitivity analysis using Complete-case Generalized Estimating Equation Models**

**eTable 4. Sensitivity Analysis using Generalized Estimating Equation Models Stratified by Sex**

**eTable 5. Sensitivity Analysis using Generalized Estimating Equation Models with Independent Correlation Structure**

**eTable 6. Sensitivity Analysis using Additionally Adjusted Generalized Estimating Equation Models**

**eTable 7. Sensitivity Analysis using Generalized Estimating Equation Models Restricted to Oxytocin as the Sole Labor Induction Agent**

**Author information**

**eMethods**

**Autism and Neurodevelopmental Assessment**

Autism spectrum disorder (ASD) was assessed using an annual questionnaire in which the participant's guardians were asked whether the child had been diagnosed with ASD, including Asperger syndrome, pervasive developmental disorder, or autism. ASD status was determined based on yes-no responses to this question.

The Ages & Stages Questionnaires, Third Edition (ASQ-3) was used in this study for neurodevelopmental screening.^1^ The ASQ-3 includes 21 guardian questionnaires to assess communication, gross motor, fine motor, problem-solving, and Personal-social characteristic domains for children aged 1–66 months.^1^ Each domain contains six questions, with parents or primary guardians scoring their child's abilities as “yes” (10 points), “sometimes” (5 points), or “not yet” (0 points), resulting in domain scores ranging from 0 to 60, where higher scores indicate better development.^1^ While the ASQ-3 is not specifically an autism screening tool, scores for each domain below the cut-off may indicate delays associated with autism and other developmental disorders. Developmental delay is defined as an ASQ-3 score below 2 standard deviations from the mean in any domain.^1^ The ASQ-3 has demonstrated robust reliability and moderate to high sensitivity in identifying developmental delays.^2,3^ The Japanese translation of the ASQ-3 has been validated, using cut-off scores for Japanese children.^4^ The ASQ-3 scores in the present study were obtained from the Japan Environment and Children's Study for children aged 36 months, 48 months, 3 years, and 4 years.

**eTable 1. Neurodevelopmental Milestones and Validated Cutoff Values in ASQ-3 Domains**

| **Domains** | **Focus** | **3 years of age** | **4 years of age** |
| --- | --- | --- | --- |
| Communication | Assessment of expressive and receptive language abilities and comprehension | 29.95 | 33.05 |
| Gross motor | Evaluation of large muscle coordination, postural control, and locomotion | 39.26 | 37.78 |
| Fine motor | Assessment of manual dexterity and hand-eye coordination skills | 27.91 | 30.18 |
| Problem solving | Measurement of cognitive abilities and early conceptual development | 30.03 | 33.87 |
| Personal-social | Evaluation of self-help abilities and interpersonal interactions | 29.89 | 35.01 |

Abbreviations: ASQ-3, Ages and Stages Questionnaires, Third Edition

**eTable 2. E-value Analysis for the Association of Epidural Analgesia and Oxytocin Exposure with Offspring ASD and Neurodevelopmental Milestones.**

|  | **ASD** | | **Below the cut off in ASQ-3 domains** | | | | | | | | | |
| --- | --- | --- | --- | --- | --- | --- | --- | --- | --- | --- | --- | --- |
|  |  |  | **Communication** | | **Gross motor** | | **Fine motor** | | **Problem solving** | | **Personal-social** | |
|  | **E-value (Point Estimate)** | **E-value (Limit of 95% CI)** | **E-value (Point Estimate)** | **E-value (Limit of 95% CI)** | **E-value (Point Estimate)** | **E-value (Limit of 95% CI)** | **E-value (Point Estimate)** | **E-value (Limit of 95% CI)** | **E-value (Point Estimate)** | **E-value (Limit of 95% CI)** | **E-value (Point Estimate)** | **E-value (Limit of 95% CI)** |
| **OT vs No exposure** | 1.28 | 1.00 | 1.11 | 1.00 | 1.28 | 1.00 | 1.00 | 1.00 | 1.25 | 1.00 | 1.29 | 1.00 |
| **LEA vs No exposure** | 4.29 | 2.05 | 1.41 | 1.00 | 1.30 | 1.00 | 1.21 | 1.00 | 1.59 | 1.00 | 1.45 | 1.00 |
| **LEA-OT vs No exposure** | 4.16 | 2.39 | 2.08 | 1.37 | 1.42 | 1.00 | 1.60 | 1.00 | 1.50 | 1.00 | 1.92 | 1.31 |

Abbreviations: ASD, autism spectrum disorder; ASQ-3, Ages and Stages Questionnaires, Third Edition; CI, confidence interval; No exposure, no exposure to either labor epidural analgesia or oxytocin administration during labor; OT, exposure to oxytocin administration without labor epidural analgesia during labor; LEA, labor epidural analgesia without oxytocin administration during labor; LEA–OT, both labor epidural analgesia and oxytocin administration during labor.

E-value (Point Estimate): the minimum strength of association (on the risk ratio or odds ratio) that an unmeasured confounder would need to have with both the exposure and the outcome, conditional on the measured covariates, to reduce the point estimate to the null value.

E-value (limit of 95% CI): the minimum strength of association that an unmeasured confounder would need to have with both the exposure and the outcome to move the limit of the 95% Cl closest to the null so that the null value lies within the interval.

**eTable 3. Sensitivity Analysis Using Complete-case Generalized Estimating Equation Models.**

|  | **No. (%)** | **ASD, aOR [95% CI]** | **Below the cut off in ASQ-3 domains, aOR [95% CI]** | | | | |
| --- | --- | --- | --- | --- | --- | --- | --- |
|  | 37 072  (100.0) |  | **Communication** | **Gross motor** | **Fine motor** | **Problem solving** | **Social personal skills** |
| No exposure | 29 027 (78.3) | 1 [Reference] | 1 [Reference] | 1 [Reference] | 1 [Reference] | 1 [Reference] | 1 [Reference] |
| OT | 7 269 (19.6) | 1.04 [0.75 - 1.45] | 0.90 [0.77 - 1.06] | 0.95 [0.83 - 1.09] | 1.01 [0.90 - 1.13] | 0.94 [0.82 - 1.06] | 0.98 [0.85 - 1.13] |
| LEA | 272 (0.7) | 2.15 [1.01 - 4.59] | 1.01 [0.62 - 1.64] | 0.87 [0.58 - 1.32] | 1.02 [0.71 - 1.47] | 1.07 [0.73 - 1.56] | 0.99 [0.64 - 1.53] |
| LEA-OT | 504 (1.4) | 2.27 [1.25 - 4.13] | 1.09 [0.76 - 1.58] | 0.99 [0.72 - 1.36] | 1.13 [0.86 - 1.50] | 1.10 [0.80 - 1.50] | 1.13 [0.81 - 1.59] |

Adjusted for geographic area during the early pregnancy period, maternal age at birth, gravidity, parity, body mass index before pregnancy, mode of delivery, duration of labor, infertility treatment, hypertension, diabetes mellitus, psychiatric disorders, hypertensive disorders of pregnancy, gestational diabetes mellitus, perinatal complications, maternal education level, marital status, maternal smoking status, maternal alcohol consumption, annual household income, paternal education level, paternal smoking status, children's sex, gestational age at birth, birth weight, 5-min Apgar score, and nursery attendance at 1 year.

Abbreviations: ASD, autism spectrum disorder; ASQ-3, the Ages and Stages Questionnaires, Third Edition; aOR, adjusted odds ratio; No exposure, no exposure to either labor epidural analgesia or oxytocin administration during labor; OT, exposure to oxytocin administration without labor epidural analgesia during labor; LEA, labor epidural analgesia without oxytocin administration during labor; LEA–OT, both labor epidural analgesia and oxytocin administration during labor.

**eTable 4. Sensitivity Analysis using Generalized Estimating Equation Models Stratified by Sex.**

| **Male** | **No. (%)** | **ASD, aOR [95% CI]** | **Below the cut off in ASQ-3 domains, aOR [95% CI]** | | | | |
| --- | --- | --- | --- | --- | --- | --- | --- |
|  | 25 717  (100.0) |  | Communication | Gross motor | Fine motor | Problem solving | Social personal skills |
| No exposure | 20 112 (78.2) | 1 [Reference] | 1 [Reference] | 1 [Reference] | 1 [Reference] | 1 [Reference] | 1 [Reference] |
| OT | 5 056 (19.7) | 1.03 [0.72 - 1.47] | 0.92 [0.77 - 1.10] | 1.07 [0.90 - 1.25] | 1.00 [0.89 - 1.13] | 0.88 [0.76 - 1.02] | 0.98 [0.84 - 1.15] |
| LEA | 192 (0.7) | 2.30 [1.03 - 5.13] | 1.11 [0.62 - 1.99] | 1.09 [0.63 - 1.88] | 0.99 [0.64 - 1.53] | 1.21 [0.77 - 1.90] | 0.96 [0.57 - 1.62] |
| LEA-OT | 357 (1.4) | 2.67 [1.31 - 5.43] | 1.33 [0.88 - 2.02] | 1.17 [0.78 - 1.74] | 1.23 [0.90 - 1.69] | 1.18 [0.82 - 1.69] | 1.44 [1.00 - 2.06] |
| **Female** | **No. (%)** | **ASD, aOR [95% CI]** | **Below the cut off in ASQ-3 domains, aOR [95% CI]** | | | | |
|  | 24 921  (100.0) |  | Communication | Gross motor | Fine motor | Problem solving | Social personal skills |
| No exposure | 19 351 (77.7) | 1 [Reference] | 1 [Reference] | 1 [Reference] | 1 [Reference] | 1 [Reference] | 1 [Reference] |
| OT | 5 007 (20.1) | 1.18 [0.57 - 2.46] | 1.02 [0.78 - 1.32] | 0.89 [0.74 - 1.08] | 1.03 [0.83 - 1.27] | 1.13 [0.92 - 1.39] | 0.91 [0.70 - 1.19] |
| LEA | 237 (1.0) | 1.22 [0.16 - 9.40] | 0.96 [0.40 - 2.29] | 0.79 [0.43 - 1.44] | 1.28 [0.68 - 2.42] | 1.11 [0.61 - 2.02] | 1.52 [0.78 - 2.95] |
| LEA-OT | 326 (1.3) | 2.67 [0.75 - 9.55] | 0.97 [0.50 - 1.89] | 0.85 [0.53 - 1.37] | 0.99 [0.57 - 1.75] | 0.94 [0.53 - 1.68] | 0.96 [0.51 - 1.81] |

Adjusted for geographic area during the early pregnancy period, maternal age at birth, gravidity, parity, body mass index before pregnancy, mode of delivery, duration of labor, infertility treatment, hypertension, diabetes mellitus, psychiatric disorders, hypertensive disorders of pregnancy, gestational diabetes mellitus, perinatal complications, maternal education level, marital status, maternal smoking status, maternal alcohol consumption, annual household income, paternal education level, paternal smoking status, children's sex, gestational age at birth, birth weight, 5-min Apgar score, and nursery attendance at 1 year.

Abbreviations: ASD, autism spectrum disorder; ASQ-3, Ages and Stages Questionnaires, Third Edition; aOR, adjusted odds ratio; CI, confidence interval; No exposure, no exposure to either labor epidural analgesia or oxytocin administration during labor; OT, exposure to oxytocin administration without labor epidural analgesia during labor; LEA, labor epidural analgesia without oxytocin administration during labor; LEA–OT, both labor epidural analgesia and oxytocin administration during labor.

**eTable 5. Sensitivity Analysis using Generalized Estimating Equation Models with Independent Correlation Structure.**

|  | **No. (%)** | **ASD, aOR [95% CI]** | **Below the cut off in ASQ-3 domains, aOR [95% CI]** | | | | |
| --- | --- | --- | --- | --- | --- | --- | --- |
|  | 50 638  (100.0) |  | **Communication** | **Gross motor** | **Fine motor** | **Problem solving** | **Social personal skills** |
| No exposure | 39 463 (77.9) | 1 [Reference] | 1 [Reference] | 1 [Reference] | 1 [Reference] | 1 [Reference] | 1 [Reference] |
| OT | 10 063 (19.9) | 1.05 [0.76 - 1.46] | 0.95 [0.82 - 1.10] | 0.99 [0.88 - 1.12] | 1.01 [0.90 - 1.13] | 0.96 [0.85 - 1.08] | 0.96 [0.84 - 1.10] |
| LEA | 429 (0.9) | 2.03 [0.96 - 4.28] | 1.06 [0.66 - 1.72] | 0.95 [0.63 - 1.42] | 1.02 [0.68 - 1.52] | 1.17 [0.82 - 1.68] | 1.12 [0.74 - 1.70] |
| LEA-OT | 683 (1.4) | 2.62^a^ [1.40 - 4.91] | 1.23 [0.86 - 1.75] | 1.03 [0.76 - 1.39] | 1.25 [0.94 - 1.67] | 1.10 [0.81 - 1.49] | 1.30 [0.96 - 1.78] |

^a^ *q*<.05

Adjusted for geographic area during the early pregnancy period, maternal age at birth, gravidity, parity, body mass index before pregnancy, mode of delivery, duration of labor, infertility treatment, hypertension, diabetes mellitus, psychiatric disorders, hypertensive disorders of pregnancy, gestational diabetes mellitus, perinatal complications, maternal education level, marital status, maternal smoking status, maternal alcohol consumption, annual household income, paternal education level, paternal smoking status, children's sex, gestational age at birth, birth weight, 5-min Apgar score, and nursery attendance at 1 year.

Abbreviations: ASD, autism spectrum disorder; ASQ-3, Ages and Stages Questionnaires, Third Edition; aOR, adjusted odds ratio; CI, confidence interval; No exposure, no exposure to either labor epidural analgesia or oxytocin administration during labor; OT, exposure to oxytocin administration without labor epidural analgesia during labor; LEA, labor epidural analgesia without oxytocin administration during labor; LEA–OT, both labor epidural analgesia and oxytocin administration during labor.

**eTable 6. Sensitivity Analysis using Additionally Adjusted Generalized Estimating Equation Models.**

|  | **No. (%)** | **ASD, aOR [95% CI]** | **Below the cut off in ASQ-3 domains, aOR [95% CI]** | | | | |
| --- | --- | --- | --- | --- | --- | --- | --- |
|  | 50 638  (100.0) |  | **Communication** | **Gross motor** | **Fine motor** | **Problem solving** | **Social personal skills** |
| No exposure | 39 463 (77.9) | 1 [Reference] | 1 [Reference] | 1 [Reference] | 1 [Reference] | 1 [Reference] | 1 [Reference] |
| OT | 10 063 (19.9) | 1.06 [0.77 - 1.48] | 0.93 [0.80 - 1.07] | 0.99 [0.87 - 1.12] | 1.00 [0.90 - 1.11] | 0.95 [0.84 - 1.07] | 0.94 [0.83 - 1.08] |
| LEA | 429 (0.9) | 2.00 [0.95 - 4.22] | 1.05 [0.65 - 1.71] | 0.95 [0.63 - 1.42] | 1.06 [0.74 - 1.52] | 1.17 [0.81 - 1.67] | 1.11 [0.73 - 1.68] |
| LEA-OT | 683 (1.4) | 2.53 [1.32 - 4.83] | 1.21 [0.85 - 1.72] | 1.04 [0.76 - 1.40] | 1.17 [0.89 - 1.55] | 1.07 [0.79 - 1.46] | 1.29 [0.94 - 1.76] |

Adjusted for geographic area during the early pregnancy period, maternal age at birth, gravidity, parity, body mass index before pregnancy, mode of delivery, duration of labor, infertility treatment, hypertension, diabetes mellitus, psychiatric disorders, hypertensive disorders of pregnancy, gestational diabetes mellitus, perinatal complications, maternal education level, marital status, maternal smoking status, maternal alcohol consumption, annual household income, paternal education level, paternal smoking status, children's sex, gestational age at birth, birth weight, 5-min Apgar score, and nursery attendance at 1 year, neonatal transport, feeding at one month, sibling cohabitation.

Abbreviations: ASD, autism spectrum disorder; ASQ-3, Ages and Stages Questionnaires, Third Edition; aOR, adjusted odds ratio; No exposure, no exposure to either labor epidural analgesia or oxytocin administration during labor; OT, exposure to oxytocin administration without labor epidural analgesia during labor; LEA, labor epidural analgesia without oxytocin administration during labor; LEA–OT, both labor epidural analgesia and oxytocin administration during labor.

**eTable 7.** **Sensitivity analysis using Generalized Estimating Equation Models Restricted to Oxytocin as the Sole Labor Induction Agent.**

|  | **No. (%)** | **ASD, aOR [95% CI]** | **Below the cut off in ASQ-3 domains, aOR [95% CI]** | | | | |
| --- | --- | --- | --- | --- | --- | --- | --- |
|  | 45 332 (100.0) |  | **Communication** | **Gross motor** | **Fine motor** | **Problem solving** | **Social personal skills** |
| No exposure | 36 417 (80.3) | 1 [Reference] | 1 [Reference] | 1 [Reference] | 1 [Reference] | 1 [Reference] | 1 [Reference] |
| OT | 7 979 (17.6) | 1.08 [0.74 - 1.56] | 0.93 [0.78 - 1.11] | 0.94 [0.81 - 1.09] | 1.00 [0.89 - 1.14] | 0.96 [0.83 - 1.11] | 0.99 [0.85 - 1.16] |
| LEA | 366 (0.8) | 2.06 [0.94 - 4.52] | 1.02 [0.60 - 1.72] | 0.97 [0.63 - 1.48] | 1.10 [0.75 - 1.62] | 1.17 [0.79 - 1.73] | 1.11 [0.71 - 1.74] |
| LEA-OT | 570 (1.3) | 2.62 [1.38 - 4.97] | 1.01 [0.67 - 1.52] | 0.92 [0.65 - 1.30] | 1.12 [0.83 - 1.52] | 1.10 [0.78 - 1.53] | 1.19 [0.84 - 1.68] |

Adjusted for geographic area during the early pregnancy period, maternal age at birth, gravidity, parity, body mass index before pregnancy, mode of delivery, duration of labor, infertility treatment, hypertension, diabetes mellitus, psychiatric disorders, hypertensive disorders of pregnancy, gestational diabetes mellitus, perinatal complications, maternal education level, marital status, maternal smoking status, maternal alcohol consumption, annual household income, paternal education level, paternal smoking status, children's sex, gestational age at birth, birth weight, 5-min Apgar score, and nursery attendance at 1 year.

Abbreviations: ASD, autism spectrum disorder; ASQ-3, Ages and Stages Questionnaires, Third Edition; aOR, adjusted odds ratio; No exposure, no exposure to either labor epidural analgesia or oxytocin administration during labor; OT, exposure to oxytocin administration without labor epidural analgesia during labor; LEA, labor epidural analgesia without oxytocin administration during labor; LEA–OT, both labor epidural analgesia and oxytocin administration during labor.

**Author information**

A list of authors and their affiliations appears at the end of the paper.

**Authors and Affiliations**

**Department of Occupational and Environmental Health, Graduate School of Medical Sciences, Nagoya City University, 1 Kawasumi, Mizuho-cho, Mizuho-ku, Nagoya 467-8601, Japan**

Asuka Tachi, Yuki Ito, Sayaka Kato & Michihiro Kamijima

**School of Nursing, Kyoto Prefectural University of Medicine, 465 Kajiichō, Kawaramachi-dōri Hirokoji-agaru, Kamigyō-ku, Kyoto, Japan**

Yuki Takahashi

**Department of Obstetrics and Gynecology, Graduate School of Medical Sciences, Nagoya City University, 1 Kawasumi, Mizuho-cho, Mizuho-ku, Nagoya 467-8601, Japan**

Mayumi Sugiura-Ogasawara

**Department of Pediatrics and Neonatology, Graduate School of Medical Sciences, Nagoya City University, 1 Kawasumi, Mizuho-cho, Mizuho-ku, Nagoya 467-8601, Japan**

Shinji Saitoh

**National Institute for Environmental Studies, Tsukuba, Ibaraki, Japan**

Shin Yamazaki

**National Center for Child Health and Development, Tokyo, Japan**

Maki Fukami

**Hokkaido University, Sapporo, Hokkaido, Japan**

Reiko Kishi

**Tohoku University, Sendai, Miyagi, Japan**

Chiharu Ota

**Fukushima Medical University, Fukushima, Japan**

Koichi Hashimoto

**Chiba University, Chiba, Japan**

Kenichi Sakurai

**Yokohama City University, Yokohama, Kanagawa, Japan**

Shuichi Ito

**University of Yamanashi, Chuo, Yamanashi, Japan**

Ryoji Shinohara

**University of Toyama, Toyama, Japan**

Hidekuni Inadera

**Kyoto University, Kyoto, Japan**

Takeo Nakayama

**Osaka University, Suita, Osaka, Japan**

Ryo Kawasaki

**Hyogo Medical University, Nishinomiya, Hyogo, Japan**

Yasuhiro Takeshima

**Tottori University, Yonago, Tottori, Japan**

Hideki Nagashima

**Kochi University, Nankoku, Kochi, Japan**

Narufumi Suganuma

**University of Occupational and Environmental Health, Kitakyushu, Fukuoka, Japan**

Mayumi Tsuji

**Kumamoto University, Kumamoto, Japan**

Kimitoshi Nakamura

**eReferences**

1. Squires, J., Twombly, E., Bricker, D., Potter, L. ASQ-3: Ages & Stages Questionnaires User's Guide. 3rd ed. Baltimore, MD: Paul H. Brookes Publishing Co; 2009.

2. Sheldrick, R. C., Marakovitz, S., Garfinkel, D., Carter, A. S., Perrin, E. C. Comparative accuracy of developmental screening questionnaires. JAMA Pediatr. 174(4), 366-374 (2020)

3. Schonhaut, L., Armijo, I., Schonstedt, M., Alvarez, J., Cordero, M. Validity of the ages and stages questionnaires in term and preterm infants. Pediatrics. 131(5), e1468-e1474 (2013)

4. Mezawa, H., et al. Psychometric profile of the Ages and Stages Questionnaires, Japanese translation. Pediatr Int. 61(11), 1086-1095 (2019)
